# Temperamental Traits Versus Individual Physical Fitness Components and a Physical Activity Level

**DOI:** 10.1515/hukin-2015-0049

**Published:** 2015-07-10

**Authors:** Dominik Bernatowicz, Paweł Izdebski, Tomasz Boraczyński, Michał Boraczyński

**Affiliations:** 1 Institute of Psychology, Kazimierz Wielki University in Bydgoszcz, Poland.; 2 Central Research Laboratory, Józef Rusiecki Olsztyn University, Poland.

**Keywords:** personality, regulative theory of temperament, physical activity, physical fitness

## Abstract

The main aim of the study was to examine whether relationships exist between particular temperamental traits within the concept of Regulative Theory of Temperament and components of physical fitness, that are most crucial for success in sport. The research involved 108 individuals including 63 men (age 21.1 ± 1.6 yrs) and 45 women (age 20.7 ± 1.3 yrs). None of the respondents were professionally engaged in sport. Components of physical fitness included: aerobic capacity, strength, agility, static-dynamic balance and reaction time. The respondents also completed two questionnaires: the Formal Characteristics of Behaviour – Temperament Inventory and the International Physical Activity Questionnaire. The results indicate that the temperamental traits had average to poor correlations with the components of physical fitness, whereas more statistically significant correlations were observed in women. Negative correlations were obtained between emotional reactivity and agility, which was a result confirmed by previous research. All temperamental traits related with the energetic aspects of behaviour correlated with simple reaction time in women. Physical activity and aerobic capacity did not correlate with any of the studied traits. The results do not allow for any general conclusions to be drawn, but can serve as a reference point for future research on temperamental traits as delineated by Regulative Theory of Temperament and their relationship with the components of physical fitness.

## Introduction

A great deal of research in the field of sport psychology is devoted to the characteristics of personality and temperament of athletes. Research on athletes’ personality and temperament, apart from descriptive purposes, aims at helping to answer the question whether there exists the phenomenon of result-oriented selection, meaning that there is a greater probability of success among individuals who are equipped with a certain set of traits. In order to find answers to these problems, correlative research seems useful in that it can compare personality and temperamental traits with achievement in sport. An original approach to this topic may be through quantifying achievement in sport by a common denominator such as the basic components of physical activity. Providing that there exists evidence that sport is practiced by individuals holding a certain set of traits (Eysenck et al., 1982; Rhodes and Smith, 2006), it seems reasonable to take a closer look at the relationship between temperamental traits and such components of physical fitness as aerobic capacity, strength, agility, static-dynamic balance and reaction time. Such a notion has not been widely described in the literature.

The theoretical basis for the present study lies in Strelau’s Regulatory Theory of Temperament (RTT), defining temperament as a group biologically conditioned, relatively constant personality traits. They can be expressed as energetic and temporal characteristics of behavior. Those temperamental traits associated with the energetic domain of reactions and behaviours include sensory sensitivity (SS), emotional reactivity (ER), endurance (EN) and activity (AC), whereas those representing the temporal dimension have been labeled as briskness (BR) and perseveration (PE) (Strelau and Zawadzki, 1993).

The structure of these six traits has been formally defined by Strelau. Sensory sensitivity is the ability to react to sensory stimuli of low stimulative value. Emotional reactivity is understood as the tendency to react intensively to emotion-generating stimuli, expressed as high emotional sensitivity with low emotional endurance. The trait of endurance involves the individual’s resistance to fatigue, determined by the ability to perform in prolonged, demanding situations without or in spite of fatigue as well as individual resistance to distractions such as those in highly stimulative conditions, i.e. when in pain. Activity is a trait that defines the tendency to undertake behaviours of a high stimulative value or to supply, by means of behaviour, strong stimulation from one’s surroundings. Briskness is the tendency to react quickly, to keep a high tempo in activities and to shift easily in behaviour (i.e. to react) as based on situational demands. Perseveration is treated as the tendency to continue and to repeat behaviour when the stimuli (situations) eliciting this behaviour are no longer present (Strelau and Zawadzki, 1993).

The biological mechanisms underlying temperament have been explained by the properties of neurons. For example, those traits related with the energetic aspects of behaviour are associated with such phenomena as the sensitivity of neuronal postsynaptic receptors or the amount of neurotransmitters released during synaptic transmission. The properties of neurons connected with the temporal aspects of behaviour include neurophysiology mechanisms responsible for the amplitude, duration, and shape of neural processes (Strelau, 1994). Netter (1991) also suggested that some neurotransmitters, such as dopamine, affected the temporal characteristics of behaviour. Of particular importance in the development of temperamental traits are the properties of nerve cells located in the cerebral cortex, reticular activating system, and limbic system (Zuckerman, 1991). This has allowed for the conclusions that the traits of temperament are a possible indicator of strength and conduction velocity of nerve impulses.

A similar viewpoint to the one outlined above was also represented by Pavlov and his disciples. In his work, Pavlov believed that the most important property of temperament was the strength of the nervous system, which in effect determines the functioning of neurons (Strelau, 1997). His disciples (Merlin and Vyatkin) later demonstrated that a ‘strong’ nervous system is most effective in sporting activities (as cited by: Strelau, 1997).

Physical fitness is directly connected with skeletal muscle activity, whereby muscular work is stimulated by the transmission of nerve impulses by motor neurons (Silverthorn, 2010). In view of this fact, it is questioned whether the temperamental traits as delineated by the RTT, themselves strongly associated with the properties of neurons, correlate with physical fitness. A review of the literature revealed that only a few studies have evaluated the above topic. One study by Guszkowska and Rychta (2007) found that the traits of temperament poorly correlate with physical fitness. Among those correlations, the majority were between the examined measures of physical fitness and emotional reactivity and activity and a greater number of correlations were found in men than in women.

The aim of this study was to explore the relationship between temperament traits with physical fitness what is justified by biological basis of these traits.

## Material and Methods

### Participants

The research involved 108 subjects including 63 men (age 21.1 ± 1.6 yrs, body mass 78.3 ± 10.6 kg, body height 1.81 ± 0.06 m) and 45 women (age 20.7 ± 1.3 yrs, body mass 61.9 ± 11.4 kg, body height 1.67 ± 0.06 m). The respondents were second-year students from the physiotherapy or tourism and recreation departments. The study was approved by the Psychology Research Ethics Committee at the Kazimierz Wielki University. All participants declared they were healthy and able to perform a physical fitness test. None of the participants were professionally engaged in sport. The exclusion criteria were contraindications for physical activity and lack of approval for participation in the research.

### Measures

The components of physical fitness that were tested included: aerobic capacity, strength, agility, static-dynamic balance and reaction time. The level of the aerobic capacity was estimated with the PWC_170_ (Physical Working Capacity) test conducted on a Monark 828 E cycloergometer. Strength abilities were estimated by measuring peak torque of the flexors of the elbow joints in static conditions with the use of a RSC-2 stabilization frame and the ZPS-3 strength measuring set (JBA-Staniak, Poland). Isometric hand strength was measured with the use of a DR4-P hand dynamometer (JBA-Staniak, Poland). To evaluate agility the 10 × 5 m shuttle run test was used (Eurofit, Council of Europe, 1993). Body balance was assessed by the Ellipsis static-dynamic test performed on a PLA2-4P tensometric platform, integrated with a WTM5 tensometric amplifier and computer software KKD v. 1.1 (JBA-Staniak, Poland). The subjects performed all tests three times. Mean error was recorded as the value of variable as a function of time between the difference of the model curve course and the course of the imaging curve.

The first questionnaire completed by the respondents was the FCB-TI (Formal Characteristics of Behaviour – Temperament Inventory, Strelau and Zawadzki, 1995) used for measuring the postulatory traits of the RTT. The test comprises 6 scales corresponding to the traits of temperament. The FCB-TI is a tool consisting of 120 test items, 20 for each of the scales.

Cronbach’s alpha as a measure of reliability was calculated separately for each scale equaling: BR – 0.77, PE – 0.79, SS – 0.73, ER – 0.83, EN – 0.85, AC – 0.83. The second test administered was the Polish version of the IPAQ (International Physical Activity Questionnaire). The IPAQ was also employed in the study to determine the time devoted to physical activity during the preceding week. The results are presented in MET (1 MET - metabolic equivalent of work - a resting energy expenditure assuming oxygen uptake of 3.5 ml/min/kg of body weight, Biernat et al., 2007).

### Procedures

The experiment consisted of two parts. The first involved the physical fitness tests and the second the above-mentioned questionnaires. All physical fitness tests were conducted in a specially adapted laboratory and the participants performed their trials individually. The questionnaires were administered simultaneously for all participants in the presence of a trained interviewer, who answered all questions in regard to how the items on the questionnaires may be interpreted. No time limits were imposed.

### Statistical analyses

Statistical analysis of the data was carried with STATISTICA 10.0 software (StatSoft. Inc., USA). The relationships between the temperamental traits, the level of physical activity and the components of physical fitness were calculated separately for men and women. The Student’s *t* test for independent samples to determine statistically significant differences among the measured variables with regards to gender was applied. The hypothesis of normal distribution was rejected using the Shapiro–Wilk test. All correlations were calculated with the Spearman’s rank correlation coefficient. The level of statistical significance was set at *p* < 0.05.

## Results

A significant correlation was found between briskness and peak torque of the elbow flexors in the group of women (0.353) and isometric hand strength in men (0.257). Perseveration correlated significantly with agility in women (0.303) and with isometric hand strength in both women (−0.368) and men (−0.382). Sensory sensitivity was found to correlate significantly with static-dynamic balance in men (0.262) and with simple reaction time in women (−0.317). Statistically significant correlations were also found between emotional reactivity and agility in women (0.329) and simple reaction time in women (0.389). In turn, endurance significantly correlated with peak elbow flexor torque in men (0.262) and simple reaction time in women (−0.295). Activity significantly correlated with simple reaction time in women (−0.329) and choice reaction time in women (−0.383). Physical activity did not correlate with any of the studied temperamental traits.

Body mass in women significantly correlated with isometric hand strength (−0.418), agility (0.329) and choice reaction time (−0.376), in men with peak elbow flexor torque (−0.313) and isometric hand strength (−0.394). Body height significantly correlated with choice reaction time in both women (−0.415) and men (0.280). With physical activity, significant correlations were found between aerobic capacity (0.525) and agility (−0.345) in women, whereas in men this variable showed significant correlations with aerobic capacity (0.523), peak elbow flexor torque (0.261) and isometric hand strength (0.279).

## Discussion

The aim of this study was to further examine the relationship between the temperamental traits defined by Strelau’s Regulatory Theory of Temperament and the components of physical fitness. Both temperament and physical fitness are known to be conditioned by various biological factors, as evidenced by passing of temperament to the offspring (Oniszczenko et al., 2003) and the ability to inherit motor skills (Bouchard et al., 1997). Therefore, this study was guided by the biological and genetic basis for acquiring these traits and the general lack of research on the relationships between these phenomena. It applied Guilford’s interpretation for correlation coefficient values (1956) to examine strength of any discovered relationships.

In the present study, statistically significant correlations were found between briskness and elbow flexor peak torque in women and isometric hand strength in men, indicating a relationship between temperamental traits and strength abilities, one which had been suggested by Tolea et al. (2012). Their study found that certain personality traits (high neuroticism, low extraversion, low conscientiousness), which were less biologically determined than temperament, were associated with muscle strength and therefore a major component of physical fitness. However, the results obtained in the present study are in disagreement with those reported by Guszkowska and Rychta (2007), who found no relationships between briskness and strength capabilities. As a result, this contradiction does allow for any significant conclusions to be drawn.

Among all the studied temperamental traits, only perseveration significantly correlated with isometric hand strength in both men and women. A rise in the level of perseveration was associated with a decrease in isometric hand strength. Moreover, in both sexes isometric hand strength was found to significantly correlate with body mass, although the correlation coefficients of perseveration with isometric hand strength in men and women were lower than those recorded between hand strength and body mass. It is well known that skeletal muscle mass determines strength. Ertem et al. (2005) demonstrated significant correlations between body mass and isometric hand strength, which may therefore modify the relationship of perseveration and isometric hand strength. However, similarly as with the trait of briskness, the results of Guszkowska and Rychta (2007) did not find any relationships between perseveration and strength abilities. The lack of congruence between their results and ours does not allow for any explicit conclusions to be made about this relationship. Therefore, further research is needed to clarify the relationship between briskness and perseveration with strength abilities but in a sample of participants with similar body mass.

Only in the group of women there was a greater degree of perseveration associated with better performance in the agility test. These results are also not in accordance with those reported by Guszkowska and Rychta (2007), in which no relationship was found between perseveration and agility. However, the statistically significant correlation between agility and physical activity as found in the group of women had been previously observed in other studies (Cuenca-García et al., 2013).

Our results also pointed to a dependency in women where increased sensory sensitivity was associated with decreased simple reaction time, although the correlation coefficients between these variables were average. This can be explained by the fact that reaction time performance is the result of the speed of the perceptual and motor systems (Magill, 2011), underpinning the notion that a high sensory sensitivity allows for quicker stimuli detection/identification.

An increase in sensory sensitivity was also associated with better static-dynamic balance in men, although the correlation coefficients here were poor. Nonetheless, this relationship may be explained by the fact that maintaining balance is, among others, associated with the activity of specific receptors (Silverthorn, 2010). At the same time, sensory sensitivity has been linked to the rate at which receptors perceive stimuli (Kantor-Martynuska, 2012). In light of the above, a logical dependency may be suggested between sensory sensitivity, receptor activity and maintaining balance. More specifically, it may be assumed that these dependencies determine the influence of sensory sensitivity on static-dynamic balance. Nonetheless, in order to confirm such a hypothesis further research is required.

The obtained results also showed that an increase in emotional reactivity was connected with improved agility in the group of women, with the obtained coefficients indicating an average correlation. In this case, similar results were obtained by Guszkowska and Rychta (2007) in a group of men. Furthermore, the present research revealed that an increase in body mass correlated with a decrease in agility in women, similar to what was obtained by Guszkowska and Rychta (2007), suggesting the existence of a cause-and-effect relationship between emotional reactivity and agility.

Increased emotional reactivity also correlated with shorter simple reaction time in women at an average level based on Guilford’s criteria (1956), which may be explained by the fact that overall greater emotional reactivity was found in this sample of women. An additional correlation that was found in women includes an increase in the temperamental trait of endurance with longer simple reaction time. The correlation coefficient for this relationship was at the threshold between weak and average strength. Further research on the cause-and-effect relationship between endurance and simple reaction time is nonetheless needed.

In men, an increase in the temperamental trait of endurance was related to an increase in peak elbow flexor torque at a poor correlative strength as based on the obtained coefficients. In addition, larger body mass and physical activity were also associated with increased peak elbow flexor torque and may have modified the above relationship. However, no significant correlations were obtained between endurance and any of the other indicators of strength (hand and lower extremity) in neither men nor women.

The results also indicate that an increase in the activity trait was associated with longer simple and choice reaction times in women. The correlation coefficients for both relationships were average. Activity is understood as the tendency to undertake behaviour of high stimulative value, with Schwerdtfeger et al. (2004) finding that individuals with a high need of stimulation achieved shorter simple reaction times. In turn, Baylé et al. (2006) argue that high impulsivity is linked with shorter choice reaction times, where impulsivity and activity correlate with each other (Strelau, 2008). However, the results obtained herein are contrary to the studies cited above, making interpretation of the relationship between activity and reaction time difficult.

The average and poor correlations between the temperamental traits of the RTT and the components of physical fitness were, in part, similar to those observed by Guszkowska and Rychta (2007), although higher coefficients between these factors were obtained in our study. All four temperamental traits related with the energetic aspects of behaviour significantly correlated with simple reaction time in women, whereas physical activity and aerobic capacity did not correlate with any of the studied traits. Additionally, in contrast to the results of Guszkowska and Rychta (2007), a greater number of statistically significant correlations were noted in women. This allows for the assumption that temperament plays a greater role in determining physical fitness in women than men.

In light of the ambiguous results of the present study and the relative originality of the subject matter, additional research is needed to better examine correlative dependencies shown here in order to attest to the cause-and-effect relationships between temperamental traits and components of physical fitness.

## Figures and Tables

**Table 1:** The correlations between temperamental traits and chosen components of physical fitness and physical activity

	women						men					

	BR	PE	SS	ER	EN	AC	BR	PE	SS	ER	EN	AC

Aerobic capacity												
Maximal torque - forearm	0.353^*^										0.262^*^	
Maximal torque - lower limb												
Isometric hand strength		−0.368^*^					0.257^*^	−0.382^**^				
Agility		−0.303^*^		−0.329^*^								
Static-dynamic balance									0.262^*^			
Simple reaction time			−0.317^*^	−0.389^*^	0.295^*^	0.329^*^						
Complex reaction time						0.383^*^						
MET												

* p < 0.05;

** p < 0.01

Explanations: BR - briskness, PE - perseveration, SS - sensory sensitivity, ER - emotional reactivity, EN - endurance, AC - activity

**Table 2:** The correlation between physical characteristics, physical activity and chosen components of physical fitness

	women			men		

	Body mass	Body height	MET	Body mass	Body height	MET
	
Aerobic capacity			0.525^**^			0.523^**^
Maximal torque - forearm				0.313^*^		0.261^*^
Maximal torque - lower limb						
Isometric hand strength	0.418^**^			0.394^**^		0.279^*^
Agility	0.329^*^		−0.345^*^			
Static-dynamic balance						
Simple reaction time						
Complex reaction time	−0.376^*^	−0.415^**^			0.280^*^	

* p < 0.05;

** p < 0.01

## References

[b1-jhk-46-211] Baylé FJ, Daban C, Willard D, Bourdel MC, Olié JP, Krebs MO, Amado-Boccara I (2006). Specific pattern of attentional changes in impulsive individuals. Cognitive Neuropsychiatry.

[b2-jhk-46-211] Biernat E, Stupnicki R, Gajewski AK (2007). International Physical Activity Questionnaire (IPAQ) – Polish version. Physical Education and Sport.

[b3-jhk-46-211] Bouchard C, Malina R, Pérusse L (1997). Genetics of fitness and physical performance.

[b4-jhk-46-211] Council of Europe (1993). Committee of experts on sport research - *EUROFIT*. Handbook for the EUROFIT test on physical fitness.

[b5-jhk-46-211] Cuenca-García M, Huybrechts I, Ruiz JR, Ortega FB, Ottevaere C, González-Gross M, Moreno LA, Vicente-Rodríguez G, Molnár D, Polito A, Manios Y, Plada M, Vanhelst J, Widhalm K, Sjöström M, Kersting M, Castillo MJ (2013). Clustering in multiple lifestyle behaviors and health-related fitness in European adolescents. J Nutr Educ Behav.

[b6-jhk-46-211] Ertem K, Harma A, Cetin A, Elmali N, Yologlu S, Bostan H, Sakarya B (2005). An investigation of hand dominance, average versus maximum grip strength, body mass index and ages as determinants for hand evaluation. Isokinet Exerc Sci.

[b7-jhk-46-211] Eysenck HJ, Nias DKB, Cox DN (1982). Sport and personality. Adv Behav Ther.

[b8-jhk-46-211] Guilford JP (1956). Fundamental statistics in psychology and education.

[b9-jhk-46-211] Guszkowska M, Rychta T (2007). Relationship between physical fitness and personality traits in adolescents. Human Movement.

[b10-jhk-46-211] Kantor-Martynuska J (2012). The princess and the pea. Suggestions for the revision of sensory sensitivity in the Regulative Theory of Temperament. J Ind Diff.

[b11-jhk-46-211] Magill R (2011). Motor learning and control: concepts and applications.

[b12-jhk-46-211] Netter P, Strelau J, Angleitner A (1991). Biochemical variables in the study of temperament: purposes, approaches and selected findings. Explorations in temperament: international perspectives on theory and measurement.

[b13-jhk-46-211] Oniszczenko W, Zawadzki B, Strelau J, Riemann R, Angleitner A (2003). Genetic and environmental determinants of temperament: A comparative study based on Polish and German samples. Eur J Personality.

[b14-jhk-46-211] Panayiotou G, Vrana SR (2004). The role of self-focus, task difficulty, task self-relevance, and evaluation anxiety in reaction time performance. Motiv Emotion.

[b15-jhk-46-211] Rhodes RE, Smith NEI (2006). Personality correlates of physical activity: a review and meta-analysis. Brit J Sport Med.

[b16-jhk-46-211] Schwerdtfeger A, Getzmann S, Baltissen R (2004). Fast reducers, slow augmenters: a psychophysiological analysis of temperament-related differences in reaction time. Int J Psychophysiol.

[b17-jhk-46-211] Silverthorn DU (2010). Human physiology: an integrated approach.

[b18-jhk-46-211] Strelau J, Wachs TD, Bates JE (1994). The concepts of arousal and arousability as used in temperament studies. Temperament: Individual differences at the interface of biology and behavior.

[b19-jhk-46-211] Strelau J (1997). The contribution of Pavlov’s typology of CNS properties to personality research. Eur Psychol.

[b20-jhk-46-211] Strelau J (2008). Temperament as a regulator of behavior. After fifty years of research.

[b21-jhk-46-211] Strelau J, Zawadzki B (1993). The Formal Characteristics of Behavior – Temperament Inventory (FCB-TI): Theoretical assumptions and scale construction. Eur J Personality.

[b22-jhk-46-211] Strelau J, Zawadzki B (1995). The Formal Characteristics of Behavior – Temperament Inventory (FCB-TI): Validity studies. Eur J Personality.

[b23-jhk-46-211] Strelau J, Zawadzki B (2011). Fearfulness and anxiety in research on temperament: temperamental traits are related to anxiety disorders. Pers Indiv Differ.

[b24-jhk-46-211] Tolea MI, Terracciano A, Milaneschi Y, Metter EJ, Ferrucci L (2012). Personality typology in relation to muscle strength. Int J Behav Med.

[b25-jhk-46-211] Zuckerman M (1991). Psychobiology of personality.

